# A Novel Soybean Intrinsic Protein Gene, *GmTIP2;3*, Involved in Responding to Osmotic Stress

**DOI:** 10.3389/fpls.2015.01237

**Published:** 2016-01-08

**Authors:** Dayong Zhang, Jinfeng Tong, Xiaolan He, Zhaolong Xu, Ling Xu, Peipei Wei, Yihong Huang, Marian Brestic, Hongxiang Ma, Hongbo Shao

**Affiliations:** ^1^Jiangsu Key Laboratory for Bioresources of Saline Soils, Provincial Key Laboratory of Agrobiology, Institute of Agro-biotechnology, Jiangsu Academy of Agricultural SciencesNanjing, China; ^2^Institute of Botany, Jiangsu Province and Chinese Academy of SciencesNanjing, China; ^3^Department of Plant Physiology, Slovak Agricultural UniversityNitra, Slovakia; ^4^Key Laboratory of Coastal Biology and Bioresources Utilization, Yantai Institute of Coastal Zone Research, Chinese Academy of SciencesYantai, China

**Keywords:** soybean, *GmTIP2;3*, heterologous expression, promoter, osmotic stress

## Abstract

Water is essential for plant growth and development. Water deficiency leads to loss of yield and decreased crop quality. To understand water transport mechanisms in plants, we cloned and characterized a novel tonoplast intrinsic protein (TIP) gene from soybean with the highest similarity to TIP2-type from other plants, and thus designated *GmTIP2;3*. The protein sequence contains two conserved NPA motifs and six transmembrane domains. The expression analysis indicated that this gene was constitutively expressed in all detected tissues, with higher levels in the root, stem and pod, and the accumulation of *GmTIP2;3* transcript showed a significant response to osmotic stresses, including 20% PEG6000 (polyethylene glycol) and 100 μM ABA (abscisic acid) treatments. The promoter-GUS (glucuronidase) activity analysis suggested that *GmTIP2;3* was also expressed in the root, stem, and leaf, and preferentially expressed in the stele of root and stem, and the core promoter region was 1000 bp in length, located upstream of the ATG start codon. The GUS tissue and induced expression observations were consistent with the findings in soybean. In addition, subcellular localization showed that *GmTIP2;3* was a plasma membrane-localized protein. Yeast heterologous expression revealed that *GmTIP2;3* could improve tolerance to osmotic stress in yeast cells. Integrating these results, *GmTIP2;3* might play an important role in response to osmotic stress in plants.

## Introduction

Lack of water resources is an important factor restricting the development of agriculture. Plant growth and development depend on water uptake and transport regulation across cellular membranes and tissues. In the past, it was thought that water moved across cell membranes by free diffusion through the lipid bilayer. However, its transport is now thought to be highly and selectively regulated by aquaporins. Aquaporins (AQPs) belong to the ancient major intrinsic protein (MIP) family found in animals, microbes, and plants. Since the discovery of AQP1 (Denker et al., 1988), many aquaporin genes have been found in plants including 35 AQPs in *Arabidopsis* (Quigley et al., 2002; Boursiac et al., 2005), 31 in *Zea mays* (Chaumont et al., 2001), and 33 in *Oryza sativa* (Sakurai et al., 2005). Guo et al. (2006) further analyzed the expression and function of the rice plasma membrane intrinsic protein (PIP) gene family. Other scholars found 23 AQPs in *Physcomitrella patens* (Danielson and Johanson, 2008), 37 in *Solanum lycopersicum* (Sade et al., 2009), 66 in soybean (Zhang et al., 2013), 47 in tomato (Reuscher et al., 2013), 71 in *Gossypium hirsutum* (Park et al., 2010), and 53 in Chinese cabbage (Tao et al., 2014). Plant AQPs can be categorized into major four subfamilies based on localization and expression patterns: plasma membrane intrinsic proteins (PIPs), tonoplast intrinsic proteins (TIPs), nodulin26-like intrinsic proteins (NIPs), small and basic intrinsic proteins (SIPs) (Chaumont et al., 2001; Kaldenhoff and Fischer, 2006), and uncategorized X intrinsic proteins (XIPs) (Danielson and Johanson, 2008).

AQPS play important roles in various physiological processes in plants, such as growth, development, and response to biotic and abiotic stresses. Srivastava et al. (2015) also reviewed the versatile functions of aquaporins as molecular conduits in the plant response to abiotic stresses. For example, Guenther and Roberts (2000) isolated two major intrinsic membrane proteins from *Lotus japonicus*, named *LIMP1* and *LIMP2*. Functional analysis using the *Xenopus oocytes* system indicated that LIMP1 appeared to be a member of the TIP subfamily and LIMP2 was a nodulin 26 ortholog protein. Rodrigues et al. (2013) investigated a gene encoding a root-specific tonoplast intrinsic aquaporin (TIP) from *Eucalyptus grandis* named *EgTIP2*, whose expression was induced by PEG and mannitol treatments but was downregulated by abscisic acid, suggesting that *EgTIP2* might be involved in the eucalyptus response to drought. Wang et al. (2014) cloned and characterized a tonoplast AQP gene (*TsTIP1;2)* from the halophyte *Thellungiella salsuginea* and reported that it mediated the transduction of both H_2_O and H_2_O_2_ across the membranes and might contribute to the survival of *T. salsuginea* under multiple stresses. Ligaba et al. (2011) studied the expression patterns of 7 *MIP* genes from barley under different abiotic stresses using quantitative real-time PCR (RT-PCR), indicating that abiotic stress modulates the expression of major intrinsic proteins in barley. Zelazny et al. (2007) by using FRET imaging analysis showed that plasma membrane aquaporins could interact to regulate their subcellular localization in living maize cells. Tomato *SiTIP2;2* expressing in transgenic *Arabidopsis* could enhance the plant's tolerance to salt stress and interact with its homologous proteins SiTIP1;1 and SiTIP2;1 (Xin et al., 2014). Gao et al. (2010) overexpressed *TaNIP*, a putative aquaporin gene from wheat, and found that it could enhance salt tolerance in transgenic *Arabidopsis*. Wang et al. (2011) cloned the novel *Glycine soja* tonoplast intrinsic protein gene *GsTIP2;1*, and the overexpression of *GsTIP2;1* in *Arabidopsis* repressed/reduced tolerance to salt and dehydration stress, suggesting that *GsTIP2;1* might mediate stress sensitivity by enhancing water loss in plants.

In this study, a novel tonoplast intrinsic aquaporin from soybean, *GmTIP2;3*, was cloned and characterized. Protein structure analysis showed that *GmTIP2;3* possesses typical aquaporin characteristics, such as six transmembrane domains and NPA motifs. The expression analysis indicated that it was constitutively expressed in all tissues tested, especially in the root, stem, and pod, and exhibited responses to ABA and PEG treatments at certain time points. Subcellular localization showed it to be localized in the cell plasma membrane. The promoter activity assay demonstrated that the core sequence for this gene was 1000 bp upstream from the ATG start codon. Yeast heterologous expression revealed that *GmTIP2;3* could improve osmotic tolerance in yeast cells. Integrating these results, *GmTIP2;3* plays an important role in response to osmotic stress in plants.

## Materials and methods

### Plant materials

*Glycine max* var. Willimas 82 was selected for the experiments, which included growth of seedlings, flowering, podding, extracting total RNA for *GmTIP2;3* cloning, and tissue expression and induced expression analysis. *Lotus japonicus* was used to transfer the promoter sequence for activity testing and Arabidopsis ecotype Col-0 was used for transformation. Protoplasts were grown in a 7:2:1 (v/v/v) mixture of vermiculite:soirite:perlite under a 16-h light/8-h dark regime, and the day and night temperatures were 23°C / 20°C, respectively. The plants were watered every week.

### Gene cloning and sequence analysis

The gene primers were designed based on the full-length coding sequences, and RT-PCR (reverse transcriptase-polymerase chain reaction) was performed to isolate the genes from soybean tissues. The neighbor-joining (NJ) tree was constructed from soybean *GmTIP2;3* and from other plant TIPs based on alignment using the Clustalx and MEGA 5.0 software, and used to explore the evolutionary relationships of soybean and other plant TIPs.

### qRT-PCR analysis

Soybean samples from the seedling, flowering, and podding stages (root, stem, leaf at young seedling stage; root, stem, leaf, and flower at flowering stage; and root, stem, leaf, and pod at podding stage) were harvested and frozen in liquid nitrogen for RNA extraction. Soybean roots were collected from plants treated with PEG6000 (polyethylene glycol) for 0, 2, 4, 12, and 48 h and with 100 μ M ABA (abscisic acid) for 10, 20, 30, 45, 60, 90, and 120 min. The total RNA for all samples used in this study was isolated using TRIzol® reagent (Invitrogen) following the manufacturer's instructions and used for qRT-PCR analysis. The qRT-PCR analysis was conducted according to the method described by Zhang et al. (2013) and was repeated three times. The primers used are given in Table 1.

**Table 1:** **Primers for this study**.

**Gene**	**Forward primer 5′-3′**	**Reverse primer 5′-3′**
*GmTIP2;3* qRT-PCR	CCTTATCTATCTTCACCTCCATCT	GCCACCAGAGATGTTGGCACCA
*GmTIP2;3* GFP	CGCAAGCTTATGGGTGGCATTGCAT	CGCGGATCCAAATTCACTGGAAAGA
*GmTIP2;3* Yeast	ATGCGGCCGCATGGGTGGCATTGCAT	CGCGGATCCAAATTCACTGGAAAGA
P1	ATGTGCAGGATGATGACCAG	CATCTTCAGAAGTTTCGAG
P2	GACTCCTCCTGCGGCTGGCATTA	CATCTTCAGAAGTTTCGAG
P3	GAAATATCATAATCTTGCTTCTTGT	CATCTTCAGAAGTTTCGAG
P4	AGGAATCATTCATTAGCTTCCGGA	CATCTTCAGAAGTTTCGAG
P5	CATAGACGTAAACAACCAATGAGT	CATCTTCAGAAGTTTCGAG
P6	GTTTACTTCTTAAAATAAACAG	CATCTTCAGAAGTTTCGAG
P7	AATATTTTTTTTAACAAAACCG	CATCTTCAGAAGTTTCGAG
P8	ATTTTGAAATTCCACAACCTCTT	CATCTTCAGAAGTTTCGAG
*GmActin*	CGGTGGTTCTATCTTGGCATC	GTCTTTCGCTTCAATAACCCTA

### Subcellular localization

PCR-generated *Hind III*-*BamH1* fragments containing the open reading frame of *GmTIP2;3* were subcloned upstream of the GFP gene in plasmid pJIT166GFP. All constructs were validated by sequencing. The primers used are listed in Table 1.

*Arabidopsis* protoplasts were isolated according to Yoo et al. (2007). The CDS of *GmTIP2;3* without stop codons was used to create an in-frame fusion with GFP gene inserted into the pJIT166-GFP vector. The resulting fusion construct and an empty vector as a control (p35S::GFP) were introduced into *Arabidopsis* protoplasts by the PEG4000-mediated method (Abel and Theologis, 1994). After incubation of transformed *Arabidopsis* protoplasts for 18–24 h at room temperature, GFP signal was detected by confocal fluorescence microscopy (Zeiss, LSM510 Meta, Carl Zeiss AG).

### Promoter analysis

A 2081 bp-long region (named P1) located upstream of the ATG start codon was cloned from soybean genome DNA using primers described in Table 1, and sequence analysis was performed using the PlantCARE online software (http://bioinformatics.psb.ugent.be/webtools/plantcare/html/). To find the core promoter region of *GmTIP2;3*, seven truncated fragments (P2-P8) were cloned from P1 and transformed into *A. rhizogenes* strain K599 for GUS activity detection. Soybean hairy root transformation was performed according to the method given by Subramanian et al. (2005). The primer pairs are listed in Table 1.

### Histochemical and fluorometric GUS assay

For histochemical staining of GUS, fresh tissue samples including whole transgenic lotus plants, soybean hairy roots, and dissected leaves from positive plants that had undergone osmotic stress (20% PEG6000 and 100 μMABA), salinity (200 mM NaCl solution), and wounding for 2 h were immediately dipped into X-Gluc solution, as previously described (Jefferson et al., 1987). After overnight incubation at 37°C in the growth chamber, stained samples were bleached with 70% (v/v) ethanol, washed several times with ddH_2_O, and observed under a Zeiss Stemi 2000-C microscope, Germany.

A quantitative fluorometric GUS assay was conducted as described by Jefferson et al. (1987). The protein concentrations from a series of truncated constructs pGUSP1-P4 in transgenic soybean hairy roots were assessed by Bradford method, using bovine serum albumin (BSA) as a standard. GUS activity was normalized to the protein concentration of each sample and calculated as nmol of 4-MU per milligram of soluble protein per minute.

### Generation of transgenic *Lotus japonicus* plants

The resulting pGUS-*GmTIP2;3* promoter(p3):GUS plasmid was introduced into *Agrobacterium rhizogenes* strain K599 and used to transform small *Lotus japonicus* seedling to produce hairy roots, as in the soybean hairy root system. The hairy roots were transferred to MS medium with 0.5 mg/L 6-BA for 20 days to generate adventive buds, and 1–2 cm high regeneration seedlings without roots were transferred to 1/2 MS medium to produce roots. Finally, the whole seedlings were transferred to pots.

### Yeast transformation

The novel pYES2-GFP was reconstructed via the recombination of pYES2 and pJIT166-GFP using the same double-digestion by *Hind III* and *EcoR I*. The CDS with *Hind III* and *BamH I* digestion for the forward primer and reverse primer, respectively (Table 1), was inserted into the pYES2-GFP vector digested with the same enzymes.

The resulting pYES2-*GmTIP2;3*:GFP plasmid was introduced into *S. cerevisiae* INVSc1 strain cells using the lithium acetate method, with the empty vector pYES2-GFP as a control. *S. cerevisiae* INVSc1 strain cells transformed with the empty vector PYES2-GFP alone and with pYES2-*GmTIP2;3*:GFP were induced with galactose and spotted on the SC-Ura medium in 0, 10, 100, 1000, and 10,000-fold-dilutions, and the drought tolerance of yeast cells expressing *GmTIP2;3* was tested by 30% PEG6000 treatment for 40 h. The GFP in yeast was observed using a fluorescence microscope (Olympus BX61). All experiments were repeated three times.

### Accession number

The accession numbers of proteins used for Multiple Sequence Alignment (MSA) and phylogenetic tree analysis are as follows: SsTIP1;1 (AJ242805.1) from *Sporobolus stapfianus*, PsTIP1;1 (AJ243309.1) from *Pisum sativum*, SoTIP2;1 (AJ245953.1) from *Spinacia oleracea*, PtTIP3;2 (XM_006372585.1) from *Populus trichocarpa*, and GmTIP2;3 (XM_006582773.1) from *Glycine max* were used for MSA. MtTIP2.1 (XP_003626979.1) from *Medicago truncatula*; AtTIP4.1 (NP_180152.1), AtTIP1.3 (NP_192056.1), AtTIP5.1 (NP_190328.1), AtTIP2.3 (NP_199556.1), AtTIP2.2 (NP_193465.1), AtTIP2.1 (NP_188245.1), AtTIP1.1 (NP_181221.1), AtTIP1.2 (NP_189283.1), AtTIP3.1 (NP_177462.1), and AtTIP3.2 (NP_173223.1) from *Arabidopsis thaliana*; OsTIP1.1 (P50156.1), OsTIP1.2 (NP_001045562.1) OsTIP2.1 (NP_001047632.1), OsTIP2.2 (Q5Z6F0.1), OsTIP3.1 (NP_001064933.1), OsTIP3.2 (NP_001053371.1), OsTIP4.1 (NP_001054979.1), OsTIP4.2 (BAA92993.1), OsTIP4.3 (NP_001042500.1), OsTIP5.1 (NP_001053493.1) from *Oryza sativa*; and ZmTIP1.1 (NP_001104896.1), ZmTIP1.2 (NP_001105029.1), ZmTIP2.1 (NP_001105030.1), ZmTIP2.2 (NP_001105031.1), ZmTIP2.3 (NP_001104907.1), ZmTIP3.1 (NP_001105032.1), ZmTIP4.1 (NP_001105033.1), ZmTIP4.2 (NP_001105034.1), ZmTIP4.3 (NP_001105035.1), ZmTIP4.4 (NP_001105641.1), and ZmTIP5.1 (NP_001105036.1) from *Zea mays* were used for phylogenetic tree analysis.

### Statistical analysis

The data were analyzed by ANOVA testing using the EXCEL software. Significant differences among means were determined by the LSD at *P* < 0.05, and a–f represent the different significant levels.

## Results

### The isolation and characterization of *GmTIP2;3*

**Figure 1:**
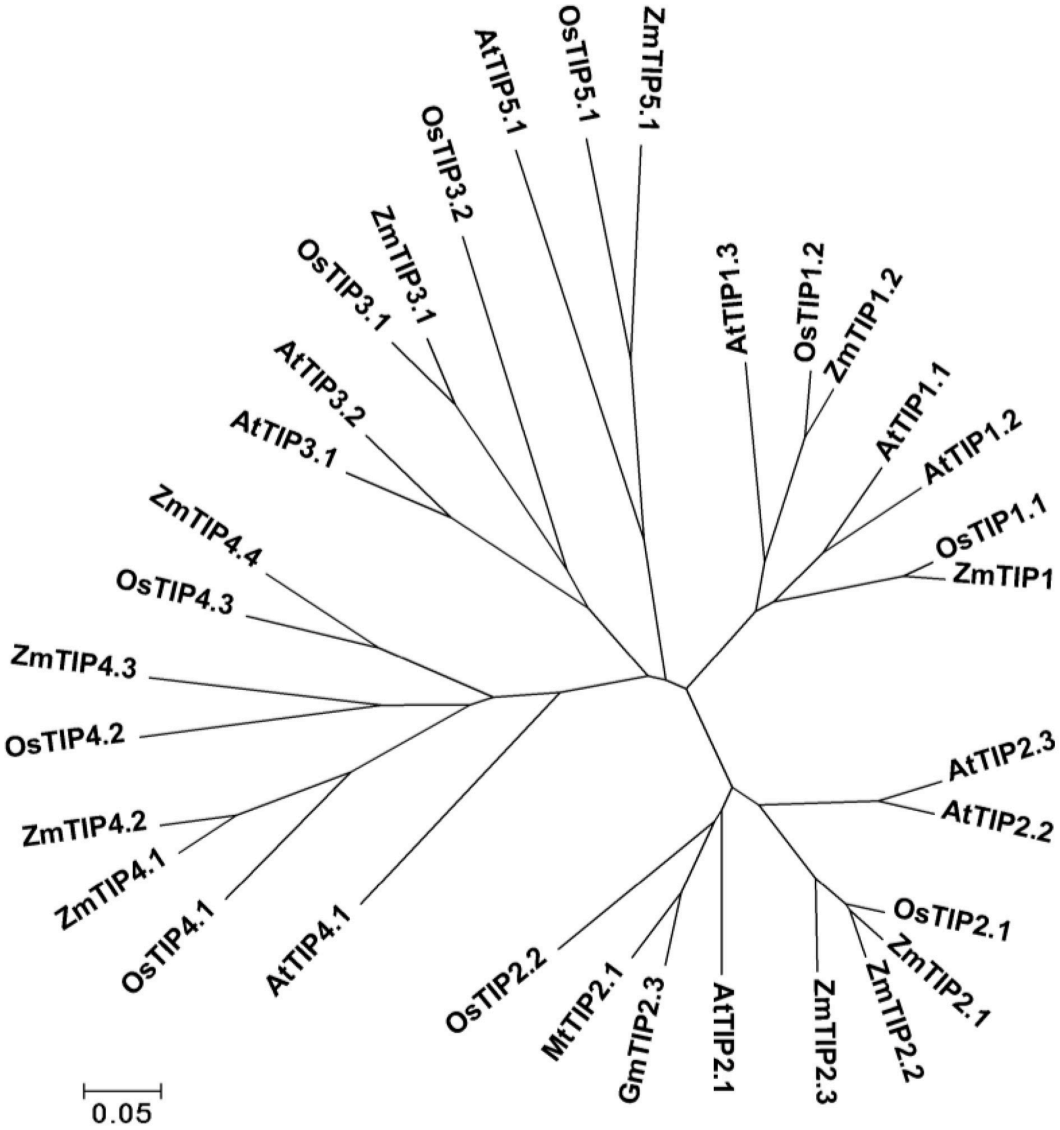
**Phylogenetic tree of ***GmTIP2;3*** and reported TIP proteins from ***Arabidopsis thaliana*** TIPs (AtTIPs), ***Oryza saliva*** TIPs (OsTIPs), ***Zea mays*** TIPs (ZmTIPs), and ***Medicago sativa*** TIPs (MtTIPs)**. The *Glycine max TIP* cloned in this paper showed the highest similarity to TIP2-Type proteins from other plants. Therefore, *TIP* was designated as *GmTIP2;3*.

**Figure 2:**
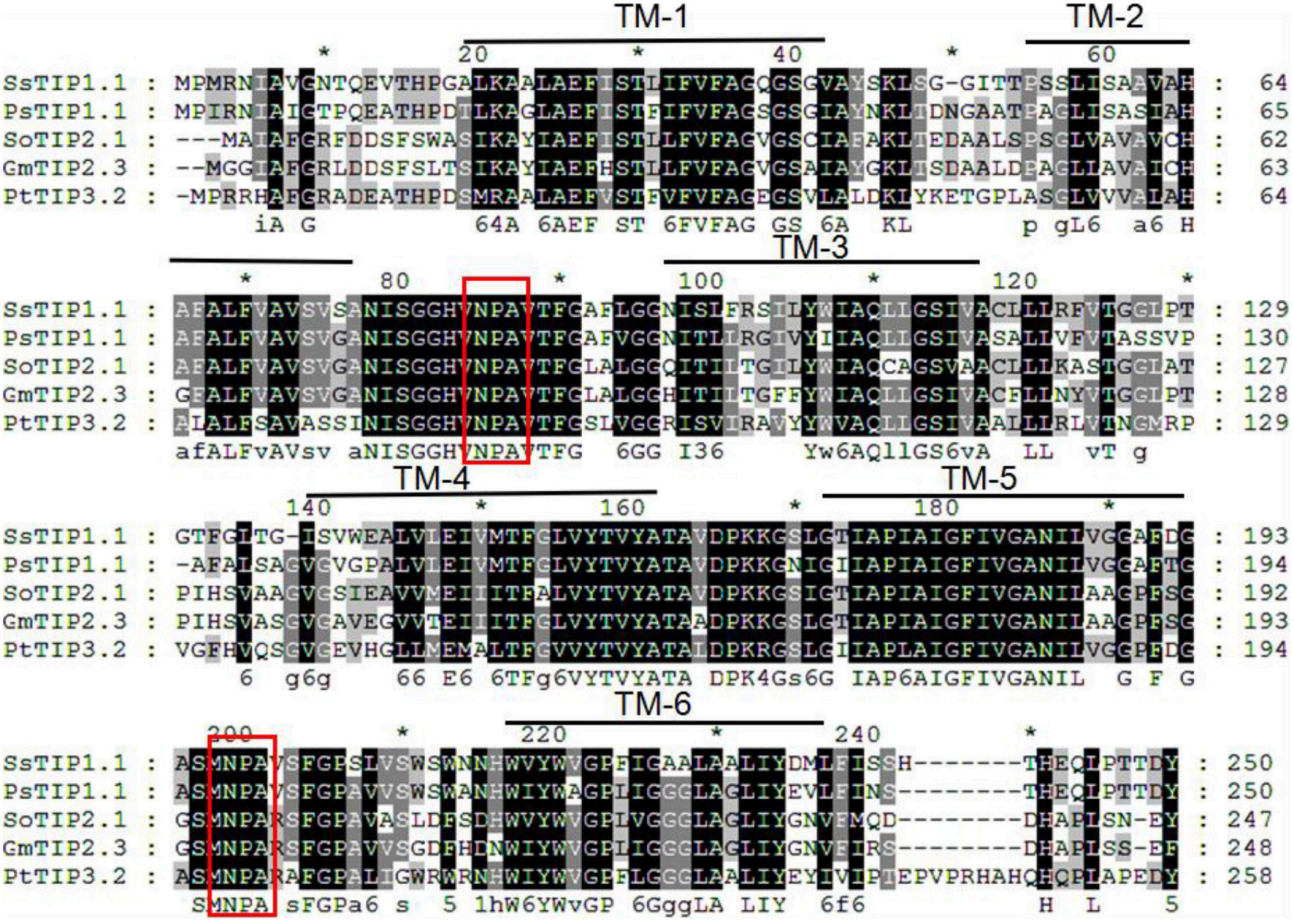
**Multiple sequence alignment of GmTIP2;3 protein amino acid sequence with other species**. TM1-6 represents the six membrane-spanning helices of GmTIP2;3. The two red boxes represent two conserved “NPA” motifs of the MIP superfamily proteins. *Indicates the consensus sequence at this site.

The gene locus Glyma07 g02060 from the QTL region between the markers Satt590 and Satt567 on chromosome 7 in soybean (Specht et al., 2001) was selected as a candidate gene and further isolated by RT-PCR method. BLAST X showed that this locus encoded a protein with 89% identity to TIP2-1-like from *Cicer arietinum.* The phylogenetic trees were created using GmTIP and *Arabidopsis thaliana* TIPs (AtTIPs), *Oryza saliva* TIPs (OsTIPs), *Zea mays* TIPs (ZmTIPs), and *Medicago sativa* TIPs (MtTIPs). The phylogenetic tree showed that GmTIP had the highest similarity to TIP2-type proteins from other plants (Figure 1). Therefore, GmTIP was designated as *GmTIP2;3*. SMART software analysis showed that its protein sequence possessed two conserved NPA motifs and six transmembrane domains, indicating that it was a typical aquaporin protein (Figure 2).

### Expression analysis of *GmTIP2;3*

**Figure 3:**
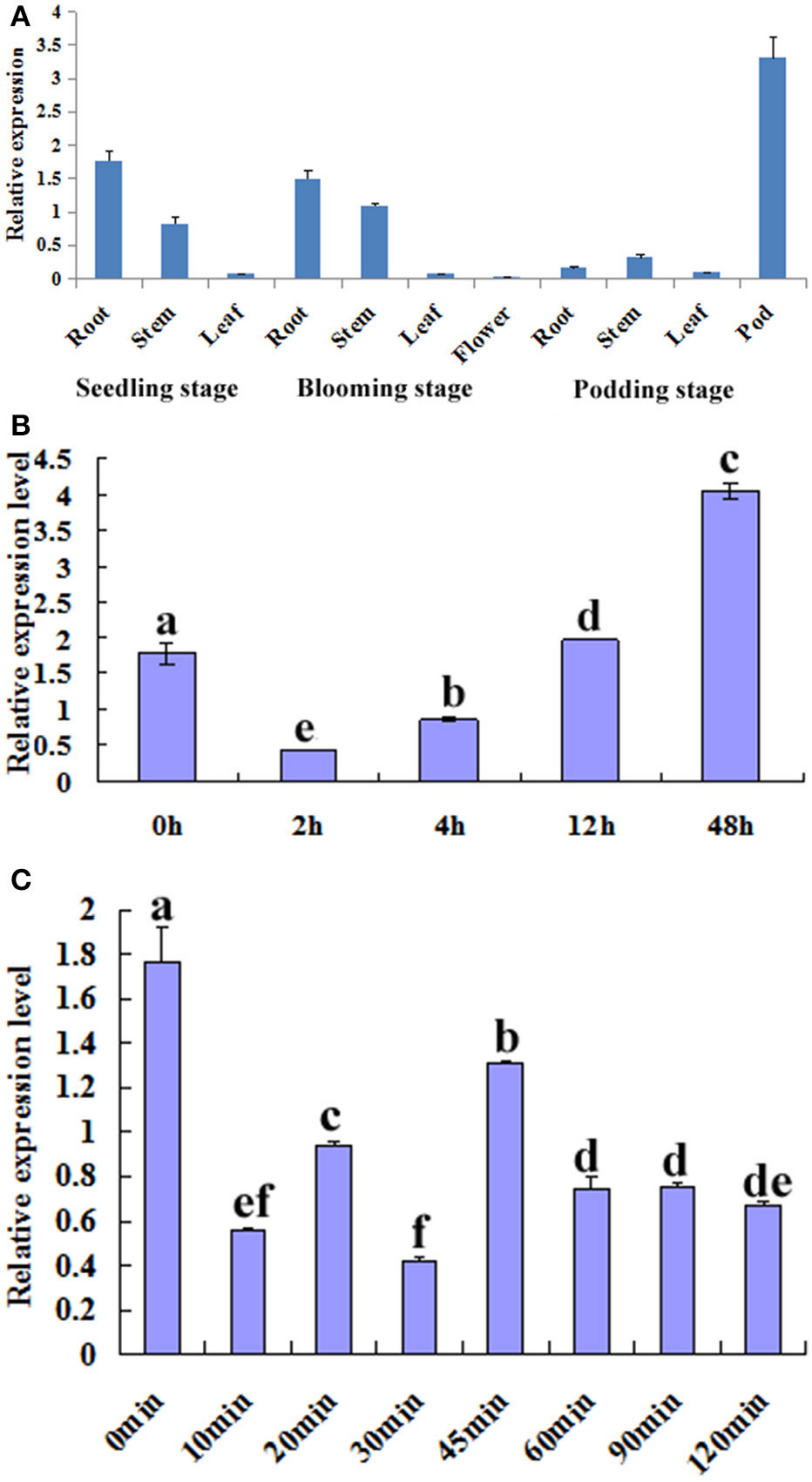
**Expression analysis of ***GmTIP2;3***.** **(A)** The temporal and spatial expression patterns of *GmTIP2;3* in different organs and at different stages. Root, stem, and leaf from three-leaf stage of young seedling; root, stem, leaf, and flower from blooming stage; and root, stem, leaf, and pod from podding stage. **(B)** The expression patterns of *GmTIP2;3* gene in soybean roots under PEG6000 and 100 μM ABA treatments; a–f indicate the significant difference level at *p* < 0.05. **(1)** Expression patterns of *GmTIP2;3* after PEG treatment for different time points. **(2)** Expression patterns of *GmTIP2;3* after ABA treatment for different time points.

The temporal and spatial expression patterns of *GmTIP2;3* in various tissues/organs of soybean cv. Willimas 82 plants were examined using quantitative RT-PCR. *GmTIP2;3* appears to be expressed in most parts of the plant, with the highest expression in the root, stem, and pod, Moreover, the expression patterns of *GmTIP2;3* in different developmental stages of the same tissue, namely in different organs in the three-leaf, blooming, and podding stages, showed that the transcript abundance of *GmTIP2;3* in the stem exhibited a slight increase at the blooming stage, then significantly decreased in both the root and stem at the podding stage except in the pod tissue (Figure 3A).

To test whether *GmTIP2;3* responds to drought stress, soybean seedling roots were treated with PEG and ABA. Then, the expression of *GmTIP2;3* was analyzed by quantitative real-time RT-PCR. The results indicated that the expression of *GmTIP2;3* decreased within 2 h after PEG-6000 (20%) treatment, and then the mRNA level continuously increased from 4 to 12 h and reached a maximum at 12 h (Figure 3B-1) However, ABA treatment (100 μM) initially significantly suppressed *GmTIP2;3* expression after 10 min treatment, reached its minimum at 30 min (*p* < 0.05), then increased from 30 to 45 min and continuously decreased from 45 to 120 min, followed by a stable expression level (Figure 3B-2).

### The promoter activity analysis of *GmTIP2;3*

**Figure 4:**
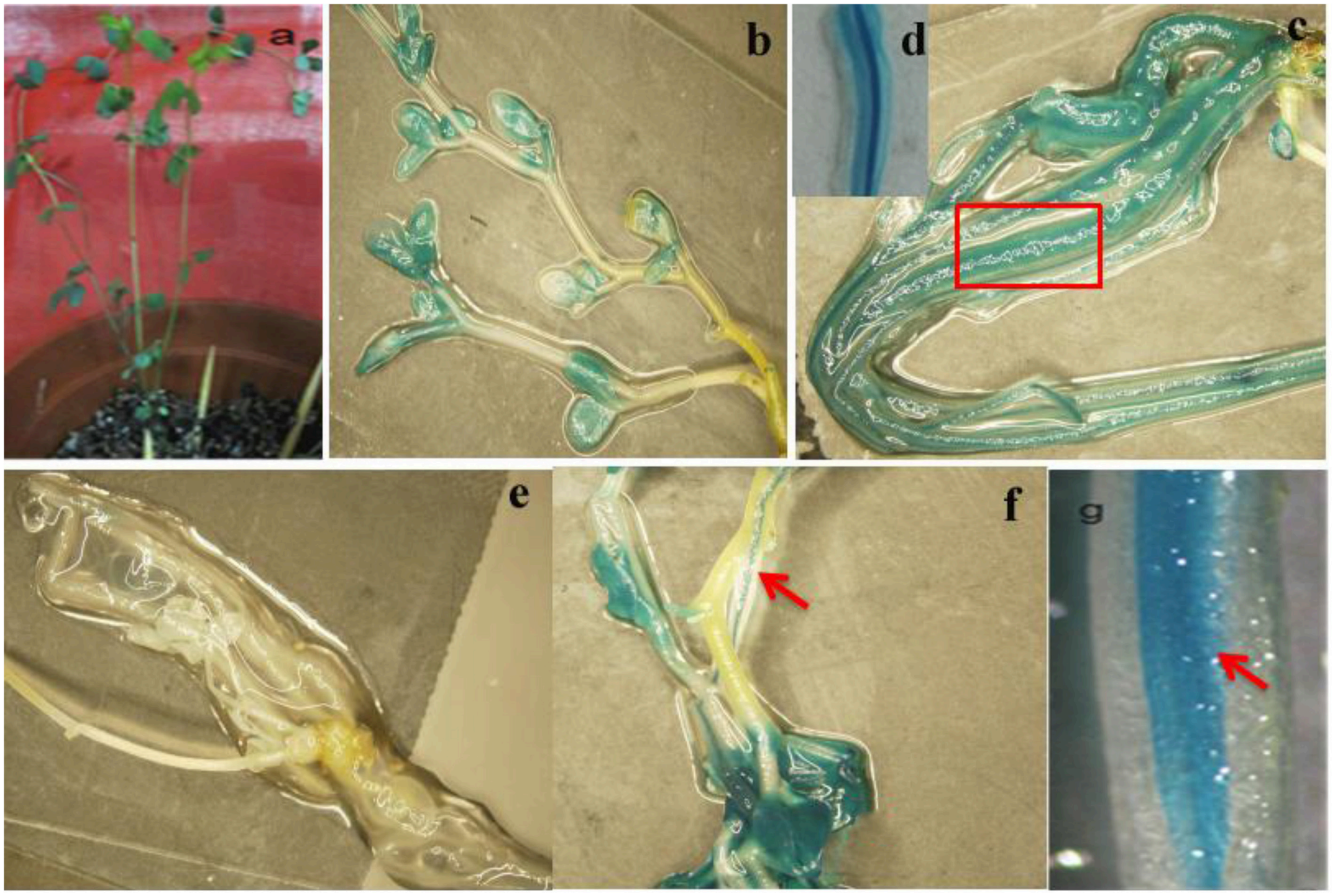
**Promoter activity analysis of ***GmTIP2;3*****. **(A)** The positive transgenic lotus plant transformed with pGUSGmTIP2;3 promoter vector. **(B)** GUS staining of transgenic positive plants. **(C)** High expression in root of positive plants. **(D)** Special expression in stele of root, magnified from root **(C)**. **(E)** GUS staining of negative plant. **(F)** High expression in leaf and stem stele of positive plant. **(G)** Special expression in stele of soybean hairy root. Red arrows indicate the stele of root and stem, and the red box indicates that this section was magnified into Panel **(D)**.

**Figure 5:**
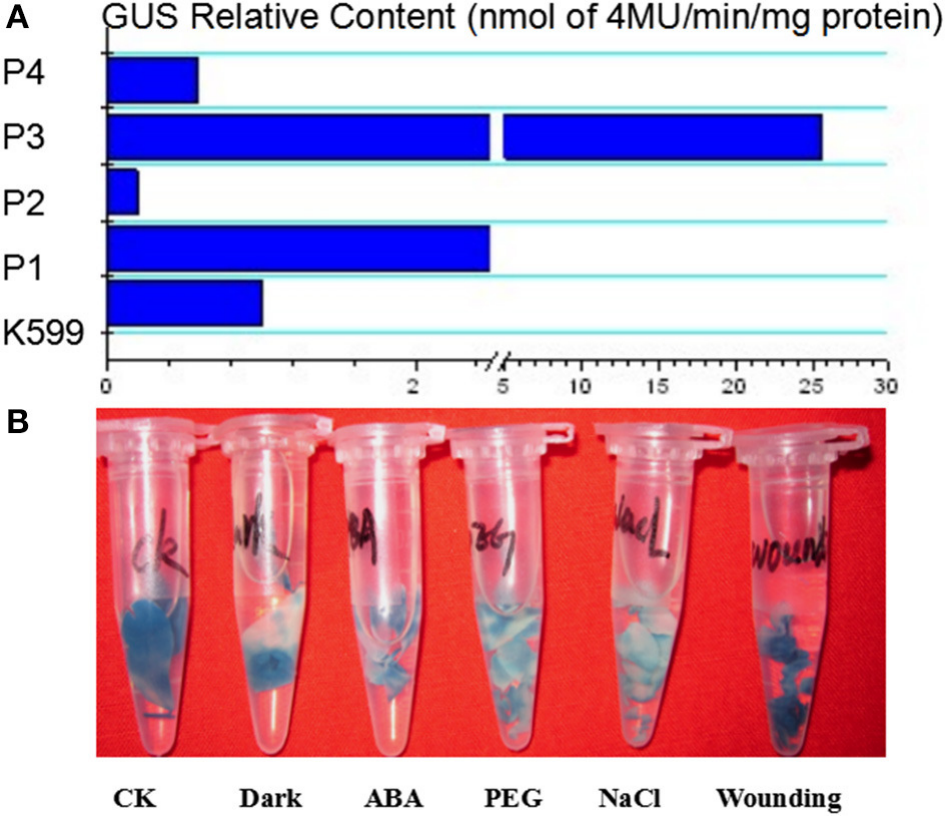
**The relative content of GUS protein harboring differently truncated promoter regions of ***GmTIP2;3*** and GUS expression patterns under different treatments using promoter 3**.

To analyze the elements contained in the promoter region and the promoter activities of GmTIP2;3, a more than 2 kb (2081 bp)-long promoter region located upstream of the ATG start codon was amplified and inserted into the pGUSP vector by the T/A cloning method. The resulting construct was transformed into *Lotus japonicus*, and the transgenic plant was successfully obtained (Figures 4A,B). The non-transgenic plant was used as a negative control (Figure 4C). GUS staining revealed that GmTIP2;3 was mainly expressed in the root (stele), stem (stele), and leaf (Figures 4D–F). Moreover, transgenic soybean hair roots using this construct also showed higher expression at the stele of the root (Figure 4G), which was consistent with its function as a water transporter. The GmTIP2;3 expression patterns in transgenic plants or hairy roots were identical to the patterns in different organs in the soybean plant. In addition, promoter sequence analysis using the PlantCARE online software indicated that it contained many light responsive elements, such as Box4, G-Box and I-Box, GATA-motif, MBS, and GARE-motif (Table 2). To further dissect the core region of the GmTIP2;3 promoter and explore the impact of external factors on its expression, a series of 8 truncated vectors were constructed, 2081, 1524, 1035, 935, 835, 735, 663, and 581 bp in length, named P1–P8, and transformed into *Agrobacterium rhizogenes* strain K599 to generate soybean hairy roots. The GUS staining and quantity assays demonstrated that only P1 and P3 exhibited GUS activities, and the activity of P3 was stronger than for P1 (Figure 5A). P5–P8 had no GUS signal, indicating that the core promoter region of GmTIP2;3 was 935 bp long from the ATG site. Interestingly, no GUS signal or GUS activity was detected for P2, implying that the inhibitor sequence occurred between P1 and P3, which also explained why the activity of P3 was stronger than for P1. Meanwhile, the expression of GmTIP2;3 was down-regulated under dark, drought (PEG and ABA), and salinity treatments for 2 h but showed no response to wounding treatment in transgenic lotus plants (Figure 5B). These results were consistent with the results of the expression patterns after treatments with ABA and PEG in soybean roots.

**Table 2:** **The cis-acting elements in ***GmTIP2;3*** promoter**.

**Site name**	**Organism**	**Position**	**Strand**	**Matrix score**	**Sequence**	**Function**
Box 4	*Petroselinum crispum*	117	+	6	ATTAAT	Part of a conserved DNA module involved in light responsiveness
Box 4	*Petroselinum crispum*	275	+	6	ATTAAT	Part of a conserved DNA module involved in light responsiveness
Box I	*Pisum sativum*	322	−	7	TTTCAAA	Light-responsive element
Box I	*Pisum sativum*	757	−	7	TTTCAAA	Light-responsive element
G−box	*Solanum tuberosum*	475	+	7	CACATGG	Cis-acting regulatory element involved in light responsiveness
GA−motif	*Arabidopsis thaliana*	1008	−	8	ATAGATAA	Part of a light-responsive element
I−box	*Zea mays*	60	−	9	gGATAAGGTG	Part of a light-responsive element
I−box	*Triticum aestivum*	1006	−	8	AGATAAGG	Part of a light-responsive element
I−box	*Flaveria trinervia*	558	−	10	cCATATCCAAT	Part of a light-responsive element
Sp1	*Oryza sativa*	353	+	6	GGGCGG	Light-responsive element
TCT−motif	*Arabidopsis thaliana*	455	+	6	TCTTAC	Part of a light-responsive element

### Plasma membrane localization of *GmTIP2;3*

**Figure 6:**
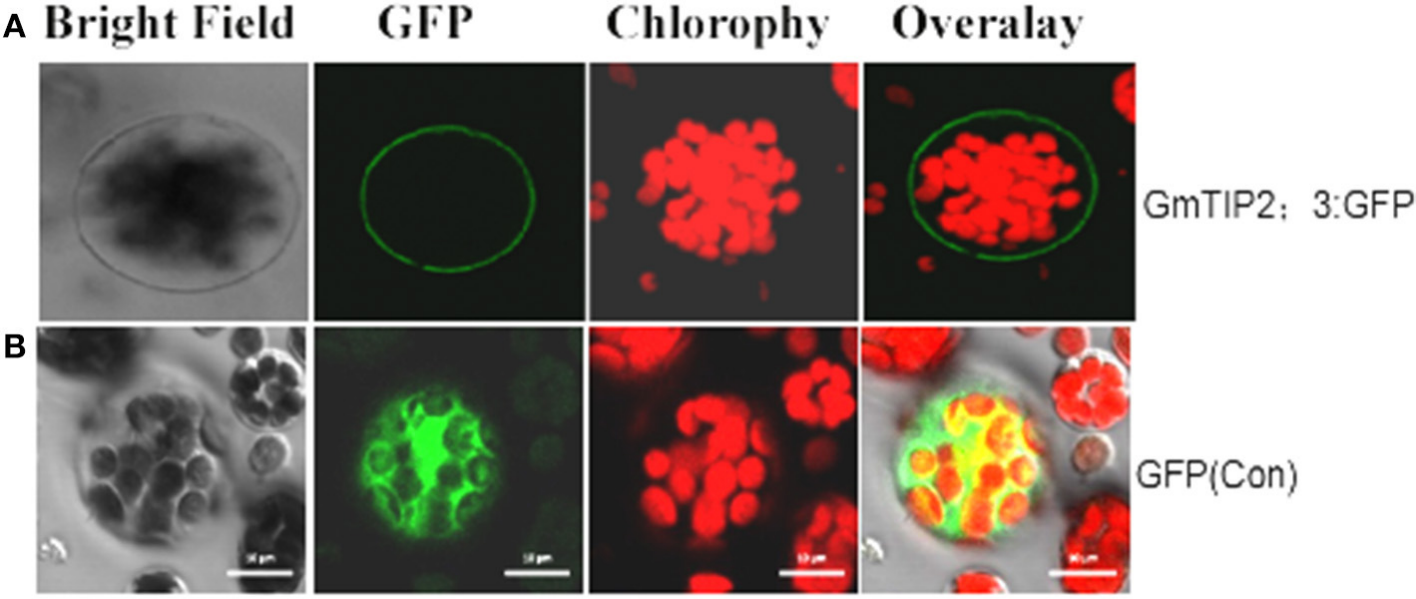
**Subcellular localization of GmTIP2;3 protein in ***Arabidopsis*** protoplasts**. The fusion construct of the GmTIP2;3-green fluorescent protein (p35S::GmTIP2;3-GFP) in the pJIT166-GFP vector without a termination codon to create an in-frame fusion between the CDS and GFP, and the GFP control plasmid (p35S::GFP), was transformed into *Arabidopsis* protoplasts by PEG4000-mediated method. The transformed *Arabidopsis* protoplasts were incubated for 18–24 h at room temperature and observed under a confocal fluorescence microscope. GmTIP2;3 was mainly located at the cell membrane **(A)**. However, the GFP control was distributed throughout the whole cell **(B)**. Scale bars = 10 μm.

To examine the localization of the GmTIP2;3 protein, the coding sequences were fused in frame with the coding region of the N-terminal side of green fluorescent protein (GFP). The fusion genes were expressed under the control of the CaMV 35S promoter. GFP fluorescence was evident in the cell plasma membrane transformed with the *GmTIP2;3*::GFP fusion plasmid (Figure 6A), whereas GFP fluorescence (control) was detected throughout the cells transformed with GFP control plasmid (Figure 6B).

### Heterologous expression *GmTIP2;3* improved osmotic stress resistance in yeast

**Figure 7:**
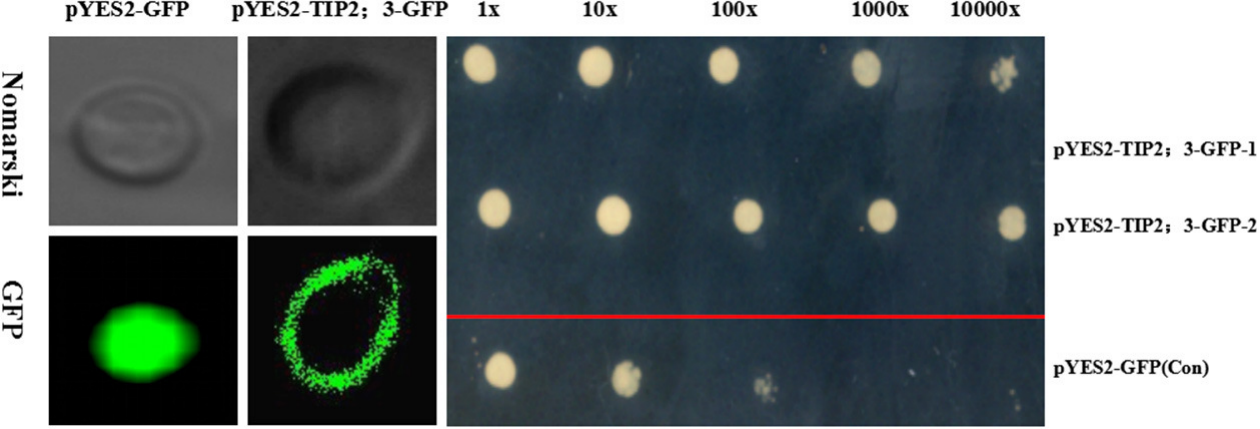
**Osmotic response of yeast cells in heterogeneously expressing ***GmTIP2;3*** S. ***cerevisiae*** INVSc1 strain cells transformed with the empty vector pYES2-GFP alone (Con) and pYES2-***GmTIP2;3***:GFP**. GFP signal indicated that GmTIP2;3 was mainly located at the cell membrane, and the empty vector was distributed throughout the cells. S. *cerevisiae* INVSc1 strain cells transformed with the empty vector pYES2-GFP alone and pYES2-*GmTIP2;3*:GFP were spotted on SC-Ura medium in 0, 10, 100, 1000, and 10,000-fold dilutions, repeated twice for two different positive clones of pYES2-*GmTIP2;3*:GFP. The results showed the osmotic tolerance of yeast cells expressing *GmTIP2;3*. All experiments were repeated three times.

Yeast cells carrying pYES2-*GmTIP2;3*:GFP or PYES2-GFP (control) were treated with PEG6000 for 40 h, and the survival state was detected. The results revealed that GmTIP2;3 was specifically expressed at the yeast cell membrane, and the heterologous expression of *GmTIP2;3* in yeast cells could improve the survival efficiency under osmotic stress (Figure 7), indicating that GmTIP2;3 played an important role in osmotic tolerance in eukaryotes.

## Discussion

In this study, we isolated and characterized GmTIP2;3, an MIP family protein showing the highest similarity to *Arabidopsis*, rice, and corn TIP5. SMART software showed that GmTIP2;3 contains six transmembrane domains, single “AEFH” and “NWIYWVGP” motifs, and two conserved NPA motifs. Fujiyoshi et al. (2002) reviewed the structure and function of water channels in mammalian aquaporins, reporting that the sequence alignment of aquaporins shows several highly conserved motifs including two “NPA” sequences and single “AEFL” and “HW[V/I][F/Y]WXGP” sequences. Here, we found that plant TIPs contain AEFI or AEFV/H, and TIPs from other plants do possess HW[V/I][F/Y]WXGP, but the soybean TIP5 had the motif NWIYWVGP, thereby implying the differences in function and localization between GmTIP2;3 and other plant TIPs.

Spatial and temporal expression analysis showed that *GmTIP2;3* was constitutively expressed in all tested organs, with higher expression in the root and stem, indicating that it can absorb water from the soil through the root and then transport water through the stem to other organs, such as the leaf, flower, and pod. Tungngoen et al. (2009) cloned and characterized two aquaporins, *HbPIP2;1* and *HbTIP1;1*, and induced expression analysis found that *HbTIP1;1* was down-regulated in liber tissues but up-regulated in laticifers in response to bark Ethrel treatment. Regon et al. (2014) also analyzed the expression patterns of 100 TIP aquaporin genes from dicots and monocots and indicated that the expression of *TIP* genes varies during different developmental stages and under stressed conditions. da Silva et al. (2013) identified and analyzed the expression patterns of sugarcane aquaporin genes under water deficit, thereby finding the aquaporin transcription in sugarcane to be potentially genotype specific. These findings demonstrated that *TIP* expression was organ specific or genotype specific and performed different regulator roles in different tissues. Recently, Lee et al. (2015) showed that the expressions of barley *HvTIP1;2* and *HvTIP3;1* were regulated by gibberellic acid (GA) and ABA and that these two hormones were involved in the fusion of protein storage vacuoles in aleurone cells, indicating that TIP plays another role in vacuole formation and transportation. When subjected to drought stress (ABA and PEG), the expression of *GmTIP2;3* showed a dynamic trend at different time points, with an increase after PEG and ABA treatments for 48 h and 45 min, respectively, indicating that the expression of *GmTIP2;3* exhibited a response to osmotic stress.

In fact, GmTIP2;3 should be a plasma membrane intrinsic protein (PIP). It was predicted to be localized at the plasma membrane by the online software http://www.predictprotein.org/, and this subcellular localization was proven using *Arabidopsis* protoplasts, yeast cells, and onion epidermal cells (data not shown) harboring GFP. However, BLAST result at NCBI showed GmTIP2;3 to be a tonoplast intrinsic protein (TIP). Analysis of the promoter activity of *GmTIP2;3* indicated that the activity of P3 (~1000 bp in length) was stronger than the activity of P1 (~2000 bp), implying that the inhibitor region occurred between these two regions, and P4 (~550 bp) exhibited no GUS activity. To further determine the core or minimum region for the *GmTIP2;3* promoter, five truncated constructs at 100 bp intervals between P3–P4 were prepared, but no GUS signal was detected. Therefore, we concluded that the core promoter region for *GmTIP2;3* was located +1000 bp upstream of the ATG start codon containing the 5′UTR region of the *GmTIP2;3* gene. The promoter–GUS system was used to detect the GUS activity changes of transgenic *Lotus* leaf under different treatments, including ABA, Nacl, dark, wounding, and PEG for 2 h. The results showed that the expression of *GmTIP2;3* decreased under all treatments except wounding. The plant CARE software revealed that the promoter region contains many light-responsive elements, so the down-regulated expression under dark conditions was reasonable. Moreover, GUS activity under drought treatment for 2 h was consistent with the expression patterns after ABA and PEG treatments for 2 h. Lee et al. (2015) detected the promoter activity of *HvTIP3;1* in response to ABA and revealed that the ABA responsiveness of the *HvTIP3;1* promoter is likely to occur via a unique regulatory system distinct from the one involving the ABA-response promoter complexes. Therefore, the mechanism of the ABA responsiveness of *GmTIP2;3* should be further examined. Here, we can hypothesize that the plants first reduce the water hole number or close water channels to reduce the loss under stress by decreasing the transcription level of *GmTIP2;3*, and then when the plants have adapted to the stress environment, the expression of *GmTIP2;3* recovers to its original level and continues to increase its transcript abundance to respond to stressed conditions.

The plant response to drought is dependent on the SPAC (Soil-Plant-Air-Continuum). Root absorption and soil play important roles in plant adaption to drought stress (Shao et al., 2009). Higher expression of aquaporin proteins in plants can allow them to effectively absorb water from the soil using the roots and then transport water by the stem to other organs, such as the leaf, flower, and seed, especially under osmotic stress (Devi et al., 2015; Ding et al., 2015; Miniussi et al., 2015; Olaetxea et al., 2015). Here, the higher expression of *GmTIP2;3* in the steles of the root and stem might promote and speed up water transportation from the roots to other organs under osmotic stress, improving plant tolerance to osmotic stress.

Azad et al. (2009) analyzed water channels by yeast heterologous expression of tulip petal plasma membrane aquaporins from *Pichia pastoris* and monitored their water channel activity (WCA) by *in vivo* spheroplast-bursting and hypo-osmotic shock assays, suggesting that *P. pastoris* can be employed as a heterologous expression system to assay the WCA and to monitor the AQP-mediating channel gating mechanism of aquaporins. The yeast heterologous expression assay in this study showed that *GmTIP2;3* could effectively improve the tolerance of yeast to drought stress. Previously, we performed this assay using salinity and drought treatments simultaneously, but the results indicated that yeast cells expressing *GmTIP2;3* did not show improved survival rates under salinity stress, implying that *GmTIP2;*3 had the ability to transport water but not ions.

### Conflict of interest statement

The authors declare that the research was conducted in the absence of any commercial or financial relationships that could be construed as a potential conflict of interest.

## Abbreviations

GFP: Green Fluorescent Protein
GUS: Glucuronidase
TIP: Tonoplast Intrinsic Protein
QRT-PCR: Quantitative reverse transcriptase Chain Reaction
CDS: Coding sequence
PEG: Polyethylene glycol
ABA: Abscisic acid.
